# Prevalence and characterization of multi-drug resistant *Salmonella* Enterica serovar Gallinarum biovar Pullorum and Gallinarum from chicken

**DOI:** 10.14202/vetworld.2016.65-70

**Published:** 2016-01-20

**Authors:** Md. Shafiullah Parvej, K. H. M. Nazmul Hussain Nazir, M. Bahanur Rahman, Mueena Jahan, Mohammad Ferdousur Rahman Khan, Marzia Rahman

**Affiliations:** Department of Microbiology and Hygiene, Faculty of Veterinary Science, Bangladesh Agricultural University, Mymensingh-2202, Bangladesh

**Keywords:** polymerase chain reaction, pulsed field gel electrophoresis, *Salmonella*, *SpeF* gene

## Abstract

**Aim:**

*Salmonella* is an important zoonotic pathogen responsible for animal and human diseases. The aim of the present study was to determine the prevalence and stereotyping of *Salmonella* isolates isolated from apparently healthy poultry. Furthermore, the clonal relatedness among the isolated *Salmonella* serovars was assessed.

**Materials and Methods:**

A total of 150 cloacal swab samples from apparently healthy chickens were collected, and were subjected for the isolation and identification of associated *Salmonella* organisms. The isolated colonies were identified and characterized on the basis of morphology, cultural characters, biochemical tests, slide agglutination test, polymerase chain reaction, and pulsed-field gel electrophoresis (PFGE). Antibiotic sensitivity patterns were also investigated using commonly used antibiotics.

**Results:**

Of the 150 samples, 11 (7.33%) produced characteristics pink colony with black center on XLD agar medium, and all were culturally and biochemically confirmed to be *Salmonella*. All possessed serovar-specific gene *SpeF* and reacted uniformly with group D antisera, suggesting that all of the isolates were *Salmonella* Enterica serovar Gallinarum, biovar Pullorum and/or Gallinarum. Antimicrobial susceptibility testing revealed that 54.54% of the isolated Salmonella Enterica serovars were highly sensitive to ciprofloxacin, whereas the 81.81% isolates were resistant to amoxycillin, doxycycline, kanamycin, gentamycin, and tetracycline. Pulsed-field gel electrophoresis of the *Xba*I-digested genomic DNA exhibited identical banding patterns, suggesting that the multidrug resistant *Salmonella* Enterica serovars occurring in commercial layers are highly clonal in Bangladesh.

**Conclusion:**

The present study was conducted to find out the prevalence of poultry *Salmonella* in layer chicken and to find out the clonal relationship among them. The data in this study suggest the prevalence of *Salmonella* Enterica, which is multidrug resistant and highly clonal for commercial layers of Bangladesh.

## Introduction

The genus *Salmonella* is phylogenically clustered in the family of *Enterobacteriaceae* [1,2]. The most *Salmonella* is motile, with the exception of the poultry-specific serovars of *Salmonella* Gallinarium and *Salmonella* Pullorum [2]. *Salmonella* is considered one of the most common causes of foodborne human infections worldwide [3]. The most characteristic feature of *Salmonella* is its wide host range, which comprises most animal species including all mammals, birds, and cold-blooded animals in addition to humans. *Salmonella* is one of the most important pathogens responsible for human food poisoning in the developed world, where chicken and chicken products are widely considered to be significant sources for this organism. Therefore, *Salmonella* has been isolated from a range of foods in almost all country in which it has been investigated [4]. Annual estimation of the incidence of nontyphoidal salmonellosis in the world is 1.3 billion, and annual death is estimated to 3 million cases [5]. The poultry population in Bangladesh is estimated to be 221.39 million of chickens and 41.23 million ducks [6]. About 50,000 chicken farms and 26,000 duck farms have already been set up in the private sector in addition to the Government farms. At present, there are more than 130 hatcheries producing 0.476 million day-old-chicks a week and about 1 million commercial layer and broiler farms supplying 0.6 million kg of poultry meat and 9.9 million table eggs per weeks [7]. However, the advancement of the poultry industry is seriously hampered due to the outbreak of various infectious and non-infectious diseases. Among the bacterial diseases, Salmonellosis is of the major problem [8]. Several environmental factors including air, dirty litter, feed, water and vectors, such as insects, humans, and rodents are responsible for *Salmonella* infection in poultry farms [9-11]. *Salmonella* infection remains as a serious problem to public health significance in worldwide and causes substantial economic loss resulting from mortality, morbidity, and poor growth with the hazard of transmitting food poisoning with gastroenteritis to human and represents a serious problem for the food industry [12] specially poultry meat. The possible source of *Salmonella* in poultry meat and eggs is due to cross contamination with feces. On the other hand, the emergence of antibiotic-resistant *Salmonella* has become a serious problem worldwide. Antimicrobials are generally applied to treat diseases as well as growth promoter in poultry, exposing a large number of birds to frequently subtherapeutic concentrations [13] and leading to the development of antimicrobial resistant pathogens that are subsequently transferred to humans through the food chain. Recently, the emergence of antibiotic-resistant *Salmonella* has also led to the ineffective treatment of salmonellosis by several ­antibiotics [14].

*Salmonella* serovars of poultry have been studied by most authors worldwide [10,11,15] and in Bangladesh [16-19]. However, most of the research works on *Salmonella* in poultry have been limited to phenotypic characterization. Data on molecular typing, such as polymerase chain reaction (PCR) and pulsed field gel electrophoresis (PFGE) are useful for epidemiological studies and is of value in identifying the potential risk to public health associated with poultry meat, eggs and other poultry products.

The aim of the present study was to determine the prevalence and stereotyping (serotypes, PCR, antimicrobial resistance patterns) of *Salmonella* isolates from apparently healthy looking poultry. In addition, the clonal relatedness among the isolated *Salmonella* serovars was assessed.

## Materials and Methods

### Ethical approval

All samples were collected as per standard sample collection procedure without harming or giving stress to any birds.

### Sample collection and processing

To isolate the *Salmonellla* spp. the samples were collected from commercial poultry farms in Mymensingh region of Bangladesh. The droppings and cloacal swabs collected from birds were transferred to the laboratory at the Department of Microbiology and Hygiene, BAU, Mymensingh. The cloacal swabs were collected in a test tube containing nutrient broth followed by incubation at 37°C for 2 h. A total of 150 samples were collected and analyzed. After taking necessary permission from the poultry farm owners, the sample collection was performed as per the guidelines set by the Department of Microbiology and Hygiene, BAU.

### Isolation and identification of Salmonella

The isolation and identification of *Salmonella* from the samples were performed according to the conventional bacteriological methods [20-24]. In short, after 2 h of incubation 10 fold dilutions was performed using nutrient broth (Himedia, India), vortexed and 0.1 ml was spread onto *Salmonella*-Shigella agar, followed by incubation at 37°C for overnight, and the plates were examined for characteristics colony produced by *Salmonella* spp.

The isolated colonies were identified and characterized on the basis of morphology, cultural characters, biochemical tests, slide agglutination test, PCR, and PFGE. The isolated organisms were identified by Gram’s staining method to determine their staining characteristics, morphology, arrangement, etc. Five basic sugar fermentation (glucose, maltose) tests were performed to identify the organism. The pure colony was identified by indole test, Methyl red, VP test and with API kit. Then the motility test was performed by hanging drop slide method and by culturing onto motility indole urea (MIU) media. Slide agglutination was performed using group D antisera (S and A Reagent Lab, Bankok, Thailand).

### Amplification of Salmonella specific gene by PCR

To perform PCR bacterial DNA was extracted by boiling method, in brief, 100 µl distilled water was taken in an eppendorf tube, a pure bacterial colony from overnight cultured was mixed with the distilled water, boiled for 10 min and then immediately cooled on ice followed by centrifugation at 10,000 rpm for 10 min. The supernatant was collected and used as DNA template for PCR.

PCR was performed using the primer pair targetting *SpeF* forward 5´-TTAGCCGTCATTGCCCGGATT-3´ and *SpeF* reverse 5´-ACGAGGTTTAATGACGTAGC-3´. For the detection of *Salmonella* spp. PCR mixture was prepared by the following method. To make 25 µl of PCR master mixture, 12.5 µl of 2 X Master mixtures (Promega, USA), 1 µl of each primer (10 pmol/µl), 4 µl of DNA template and remaining 6.5 µl deionized water were added in PCR tube. To amplify the *SpeF*, PCR reaction was performed in 25cycles with initial denaturation 92°C for 5 min, denaturation 92°C for 30 sec, annealing 50°C for 1 min, extension at 72°C for 3 min and a final extension at 72°C for 5 min. The amplified products were resolved in 1% agarose gel stained with ethidium bromide, electrophoresed at 100 volt and examined under UV transilluminator.

### PFGE

Whole agarose-embedded genomic DNA from the *Salmonella* isolates was prepared. PFGE was carried out using a contour-clamped homogeneous electrical field-DRII apparatus (Bio-Rad), according to procedures described elsewhere [25]. Genomic DNA of the test strains was digested by *Xba*I restriction enzyme (Gibco-BRL, Gaithersburg, MD), and *Salmonella* Enterica serovar Braenderup was digested using *Xba*I, with fragments employed as molecular size markers. Restriction fragments were separated in 1% pulsed-field-certified agarose in 0.5X TBE (Tris-borate-ethylenediaminetetraacetic acid) buffer. Post-electrophoresis gel-treatment included gel stained and de-stained. The DNA was visualized using a UV-transilluminator, and images were digitized via a one-dimensional gel documentation system (Bio-Rad). The fingerprint pattern in the gel was analyzed using a computer software package, Bionumeric (Applied Maths, Belgium). The fingerprint patterns were subjected to typing based on banding similarity and dissimilarity, using the dice similarity coefficient and unweighted pair group method employing average linkage (UPGMA) clustering, as recommended by the manufacturer. The results were graphically represented as a dendrogram.

### Antimicrobial susceptibility test

The antimicrobial susceptibility tests of the isolates were performed by disc diffusion method according to CLSI [26]. The antibiotic discs impregnated with ciprofloxacin (5 mcg), amoxycillin (10 mcg), doxycycline (30 mcg), kanamycin (30 mcg), gentamycin (10 mcg), tetracycline (30 mcg) (Oxoid, UK) were used in this study.

## Results

**Figure-1:**
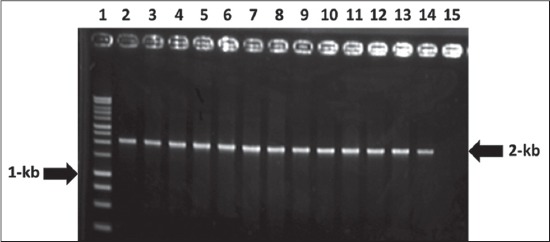
Lane 1: 1-kb DNA ladder, Lane 3-13: Tested samples positive for *SpeF* gene. Lane 2, 14 positive control, and Lane 15 negative control.

**Figure-2:**
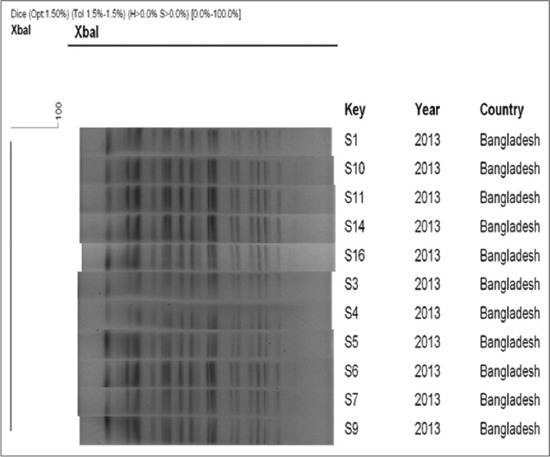
Genomic fingerprinting of *Salmonella* isolated from poultry farm of Bangladesh Agriculture University in 2013. The dendrogram was constructed by dice similarity coefficient and UPGMA clustering method using pulsed-field gel electrophoresis images of *XbaI*- restriction digested genomic DNA of the tested *Salmonella* strains; the scale bar at the top (left) indicates similarity coefficient.

In the present study out of 150 samples collected, eleven samples were identified as positive for *Salmonella* by conventional techniques, API 20E system, serotyping and molecular methods. On S-S agar the *Salmonella* isolates produced translucent, black, smooth, small round colonies. Microscopic examination of Gram stain revealed Gram-negative, pink colored short rod-shaped bacteria, arranged either single or paired. All of the test isolates were indole and V-P negative and Methyl Red test positive, fermented glucose and maltose and produced both acid and gas. The isolates were agglutinated with group D antiserum, indicated that all 11 isolates were *Salmonella* Pullorum and/or *Salmonella* Gallinarum. All isolates were found to be non-motile characterized by forming the stab line without producing turbidity in the MIU medium. All the isolated *Salmonella* were confirmed as *Salmonella* Pullorum and/or *Salmonella* Gallinarum by PCR targeting *SpeF* gene. The *SpeF* genes specific 2 kb amplicon sizes (Figure-1) were successfully amplified in all isolates. In this study out of 150 cloacal swabs, *Salmonella* Pullorum and Gallinarum were isolated from 5 (3.33%) and 6 (4.00%) birds and differntiated based on biochemical characters. The biovars *Salmonella* Gallinarum and Pullorum were differentiated based on different sugar fermentation tests particularly dulcitol fermentation test. The biovar, Gallinarum can ferment galactose and dulcitol. In our study total 6 of the biovars fermented galactose and dulcitol and 5 isolates were found non-fermenter. All 5 isolates were fermented glucose and confirmed as Pullorum. All the isolated 11 *Salmonella* spp. was subjected to PFGE to determine their clonal relatedness. The *Xba*I- restriction enzyme digested the genomic DNA and produced 16-20 fragments (Figure-2) ranged from 550 to 20-kb of molecular sizes. Cluster analysis, which was performed with dendrograms (prepared by dice similarity coefficient and UPGMA clustering methods), revealed a single cluster with 100% similarity coefficient (Figure-2). Antimicrobial susceptibility test using six drugs revealed that 54.54% of the isolated *Salmonella* Enterica serovars were highly sensitive to ciprofloxacin, whereas 81.81% isolates were resistant to amoxycillin, doxycycline, kanamycin, gentamycin, and tetracycline.

## Discussion

*Salmonella* Gallinarum and *Salmonella* Pullorum are non-motile pathogens that infect poultry and other galliform birds [27]. *Salmonella* Gallinarum is responsible for fowl typhoid, and *Salmonella* Pullorum causes Pullorum disease, which is characterized by white diarrhea in chicks [28]. SG, found mostly in adult birds, can also affect young birds. *Salmonella* Pullorum may infect older chickens, causing symptoms similar to those observed in fowl typhoid. Therefore, the proper identification of both *Salmonella* serovars is a very important from the epidemiological and control standpoints.

In the present study, we identified 7.33% *Salmonella* in healthy layer chicken. The results are in agreement with [29] in Basrah city who found that the overall presence of *Salmonella* spp. was 9.2%. On the basis of cultural, serological, biochemical and molecular characterization, i.e., the overall prevalence of *Salmonella* in a cloacal swab of healthy layers is 7.33% in Mymensingh region of Bangladesh. Several authors reported that unlike other *Salmonella* serotypes, Pullorum and Gallinarum are not excreted extensively in feces [30]. Rahaman *et al*. [30] observed that in the absence of clinical signs bacteriological assay of faecal swabs for Pullorum or Gallinarum will not be easy. The most important observation in this study was the excretion of *Salmonella* Pullorum in healthy flocks which needs to be considered when flocks are stressed due to other factors as such excretions can be a source of infection in multi-age farms. *Salmonella* Enterica serovar Pullorum and Gallinarum are worldwide a poultry pathogen of considerable economic importance, particularly a developing poultry industry. *Salmonella* Enterica serovar Pullorum colonized both the ovary and the oviduct of hens and led to 6% of laid eggs being infected by *Salmonella* serovar Pullorum. The colonization of several different sites of the reproductive tract suggests that serovar Pullorum may employ more than one mechanism of egg infection [18].

Although *Salmonella* Pullorum and *Salmonella* Gallinarum cause different diseases in poultry, they are very similar in genetically and phenotypically and it is very difficult to differentiate these two Salmonellae. *Salmonella* Gallinarum is responsible for fowl typhoid, and *Salmonella* Pullorum causes Pullorum disease, both are non-motile and contain the same somatic antigenic structure [31]. The differentiation between *Salmonella* Pullorum and *Salmonella* Gallinarum is very important from epidemiological and preventive perspectives and also for the development of vaccine. As these two organisms are very similar, and cannot be distinguished by conventional and serological methods [31]. The biovars *Salmonella* Gallinarum and Pullorum were differentiated in the study based on different sugar fermentation tests particularly dulcitol fermentation test. This biovar differentiation concurs with the report of Rahaman *et al*. [30] who differentiated biovar by the use of sugars such as maltose, dulcitol and glucose.

Antibimicrbial resistance in *Salmonella* has assumed alarming proportion worldwide [32]. It is associated with improper use of antimicrobial agent and has been reported to occur mostly in hosts that receive the antimicrobial drugs. In this study, multidrug resistance *Salmonella* were isolated might be due to wide use of antibiotics in commercial poultry farms. Antibiogram using six drugs revealed 54.54% isolates were sensitive to ciprofloxacin and 81.81% isolates were resistant to amoxycillin, doxycycline, kanamycin, gentamycin, and tetracycline. Ramya *et al*. [33] described that the sensitivity of *Salmonella* spp. was 100% for ciprofloxacin followed by amoxycillin (82%). Hyeon *et al*. [34] and Roy *et al*. [23] stated that in *Salmonella* the highest antibiotic resistance observed was to erythromycin (100%) followed by streptomycin (22.2%) and tetracycline and chloramphenicol (16.7%).

The isolation of *Salmonella* Gallinarum and *Salmonella* Pullorum from cloacal swab samples indicates the existence of fowl typhoid and Pullorum disease in intensive poultry farms in Mymensingh. All of the isolates showed identical restriction cleavage patterns in PFGE. The PFGE pattern of all the strains matched with each other in the number and position of the DNA fragments, suggesting genetic homogeneity. Pulsed-field gel electrophoresis of the *Xba*I-digested genomic DNA exhibited banding patterns that were identical (the similarity coefficient was 100%) for all the tested isolates which formed a tight cluster when dendrogram was constructed using the PFGE patterns, suggesting a high level of clonal relatedness among them. The data in this study suggest the prevalence of *Salmonella* Enterica, which is multidrug resistant and highly clonal for commercial layers of Bangladesh.

## Conclusion

*Salmonella* is regarded as the major bacterial foodborne pathogen causing human illnesses worldwide. It cannot be debated that poultry meat and eggs are major vehicles for *Salmonella* transmission to human. As such, researchers, veterinarians, processors and the government are working in tandem to search for a new method of testing and characterization for *Salmonella*. The present study was conducted to find out the prevalence of poultry *Salmonella* in layer chicken and to find out the clonal relationship among them. The data in this study suggest the prevalence of *Salmonella* Enterica, which is multidrug resistant and highly clonal for commercial layers of Bangladesh.

## Authors’ Contributions

MSP, MJ and MFRK have participated in isolation of samples and conventional identification of *Salmonella* spp., and drafting the manuscript. KHMNHN and MR have conducted the molecular identification of the bacteria. MBR and KHMNHN have critically checked the manuscript for finalization.
